# Magnetic resonance imaging (MRI) has failed to distinguish between smaller gut regions and larger haemal sinuses in sea urchins (Echinodermata: Echinoidea)

**DOI:** 10.1186/1741-7007-7-39

**Published:** 2009-07-13

**Authors:** Nicholas D Holland, Michael T Ghiselin

**Affiliations:** 1 Marine Biology Research Division, University of California at San Diego, La Jolla, CA, 92093, USA; 2 California Academy of Sciences, 55 Concourse Drive, Golden Gate Park, San Francisco, CA 94118, USA

## Abstract

A response to Ziegler A, Faber C, Mueller S, Bartolomaeus T: Systematic comparison and reconstruction of sea urchin (Echinoidea) internal anatomy: a novel approach using magnetic resonance imaging. *BMC Biol *2008, 6: 33.

## Commentary

The sea urchin siphon is a narrow-bore tube that originates near the esophagus-stomach junction, runs parallel to the stomach, and finally rejoins the main course of the gut at the beginning of the intestine. Although a siphon is present in most sea urchins, its place is taken by a siphonal groove in the following three major clades (traditionally ranked as families): Cidaridae [1], Diadematidae [2], and Pedinidae [3]. The presence of a siphonal groove in the Diadematidae recently became controversial when two publications [4,5] claimed that such sea urchins actually have a siphon instead and that our previous report to the contrary [2] was based on a "mistaken observation."

We responded to the criticism in [4,5] with a rebuttal [3] using scanning electron microscopy and histological sectioning to support our original conclusion that diadematids have a siphonal groove and not a siphon. In spite of our rebuttal, the opposing claim – that diadematids have a siphon rather than a siphonal groove – has since been perpetuated in a paper published in BMC Biology [6]. Thus the present correspondence presents additional evidence in hope of finally laying this controversy to rest. We also consider the reasons why such strikingly discordant views of sea urchin morphology could have arisen in the first place.

The authors who disagree with us [4-6] studied the sea urchin digestive tract without considering an intimately related component of the haemal system: namely the inner marginal sinus, which runs along the adaxial side of the stomach. Moreover, those authors used either gross dissection [4,5] or presented horizontal MRI sections [6], showing only the outer contours of the gut without providing any information on the internal details. Such details are indispensable for distinguishing the digestive tract, which is lined by an epithelium [7] from the haemal system, which is not [8]. Thus, the data presented in [4-6] could not distinguish the inner marginal sinus from the gut.

**Figure 1:**
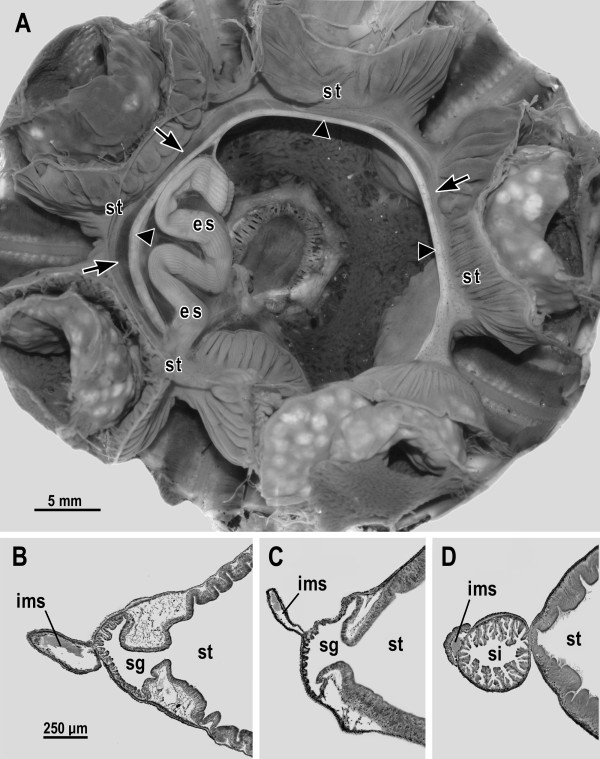
**Siphonal grooves versus siphons in sea urchins**. (A) Gross anatomical dissection of *Diadema setosum *seen from the oral side after removal of the jaw apparatus (photograph by Gregory W. Rouse). Conspicuous gut regions are the esophagus (es) and stomach (st), which makes a clockwise circuit of the body from approximately 6 o'clock to 5 o'clock. The siphonal groove (indicated by arrows) and the inner marginal sinus (indicated by arrowheads) of the haemal system accompany the stomach throughout its course. (B, C) Cross sections of the inner marginal sinus (ims), siphonal groove (sg), and part of the stomach (st) of two diadematid species: (B) *Diadema setosum *and (C) *Diadema antillarum*. (D) Cross section of the inner marginal sinus (ims), siphon (si), and part of the stomach (st) of a non-diadematid sea urchin, *Arbacia incisa*. In all the cross sections, the inner marginal sinus contains a clot of haemal fluid.

Figure 1A illustrates the features of the sea urchin gut and haemal system that are relevant for the present argument. Figure 1B–D illustrates histological sections from two diadematids (*Diadema setosum *purchased at an aquarium store and *Diadema antillarum *collected in Bimini, Bahamas) and from one sea urchin species with a siphon (*Arbacia incisa *collected near San Diego, California). The specimens, which were all approximately 5 cm in test diameter, were fixed in 10% formalin seawater, embedded in paraplast, prepared as serial sections 12 μm thick, and stained with 0.1% aqueous azure A. In diadematids, the inner marginal sinus is relatively large (Figure 1B, C) as compared to that of most other sea urchins (Figure 1D). Indeed the diameter of the inner marginal sinus in diadematids approaches the diameter of the siphon in other sea urchins. Thus, if one looks at structures exclusively by the techniques of gross dissection [4,5] and MRI [6], the inner marginal sinus of diadematids will give the impression of being the siphon [4-6]. In contrast, the present study of two species in the genus *Diadema *reveals the internal anatomy in sufficient detail (Figure 1B, C) to show unequivocally the presence of a siphonal groove and not a siphon. An identical result was obtained in our initial histological study [2] that included five diadematid species in addition to the two illustrated here. From the weight of the evidence it is safe to say that all sea urchins in the taxon Diadematidae, which is monophyletic by the analyses of [9], have a siphonal groove and not a siphon.

## Response

Magnetic resonance imaging: a powerful tool in comparative morphology despite initial interpretative difficulties

Alexander Ziegler* ^1^, Thomas Bartolomaeus^2^

^1^Institut für Immungenetik, Charité-Universitätsmedizin Berlin, Berlin, Germany

^2^Institut für Evolutionsbiologie und Zooökologie, Rheinische Friedrich-Wilhelms-Universität Bonn, Bonn, Germany

*Corresponding author

Email

AZ: alexander.ziegler@charite.de

TB: tbartolomaeus@evolution.uni-bonn.de

Drs. Holland and Ghiselin have convincingly pointed out, initially as a direct response to Drs. Campos and Moura [5], and now in the correspondence above, that Pedinidae and Diadematidae are indeed characterized by the presence of a siphonal groove rather than a siphon. Their data effectively challenge our interpretation of these structures in *Caenopedina mirabilis *(Pedinidae) and *Diadema savignyi *(Diadematidae), as presented in our BMC Biology paper [[6], Fig. 3]. The images of 3D models depicted in this article were based exclusively on datasets generated using magnetic resonance imaging (MRI). We are happy to see these points of interpretation corrected and this issue of minor controversy resolved.

**Figure 2:**
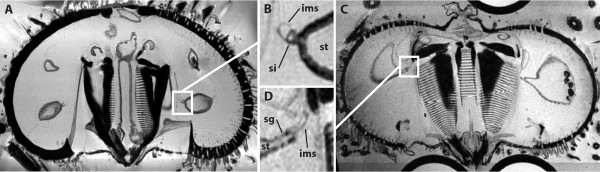
**Magnetic resonance imaging: potential and limitations for comparative morphological analyses**. Vertical MRI sections of two sea urchin (Echinoidea) species passing from pharynx to periproct. **A ***Psammechinus miliaris *(Müller, 1771), (44 μm)^3 ^dataset resolution, contrast agent: Magnevist. **B **Inset from **A **showing the stomach (st) with adjacent siphon (si) and inner marginal sinus (ims). **C ***Diadema savignyi *Michelin, 1845, (40 μm)^3 ^dataset resolution, contrast agent: Magnevist. **D **Inset from **C **showing the stomach (st) with siphonal groove (sg) and adjacent inner marginal sinus (ims). Although both scans permit to differentiate various minute and large structures, the inner marginal sinus (ims) present in *D. savignyi *can be mistaken for a siphon. Vertical sections and magnifications were generated using the Volume Viewer in ImageJ 1.41o.

We would nevertheless like to point out that the obvious advantages of non-invasive imaging techniques such as MRI or micro-computed tomography for large-scale comparative morphological analyses are not undermined by the fact that certain anatomical details are presently difficult to resolve. Currently, this opens the opportunity for erroneous designations, e.g. of a haemal structure as the siphon in Diadematidae [6] (see also Fig. 2). However, with higher resolutions as well as extended contrasting techniques, the room for such misinterpretations is likely to decrease. We are also happy to acknowledge the importance of complementary investigative techniques such as manual dissection, histology and electron microscopy, and the additional opportunities afforded by employing these in combination with MRI, as in our recent publication [10].

For some analyses, however, invasive techniques are not appropriate. Possibly, the most important attribute of modern imaging techniques is their non-invasive nature, which permits the study of rare specimens and the elucidation of the original topography of organ systems. This is of particular importance in organisms whose soft tissue structures are enclosed by skeletal elements, as is the case in sea urchins.
